# The Dynamics of Son Preference, Technology Diffusion, and Fertility Decline Underlying Distorted Sex Ratios at Birth: A Simulation Approach

**DOI:** 10.1007/s13524-016-0500-z

**Published:** 2016-09-16

**Authors:** Ridhi Kashyap, Francisco Villavicencio

**Affiliations:** 1 Max Planck Institute for Demographic Research, Rostock, Germany; 2 Nuffield College and Department of Sociology, University of Oxford, Oxford, United Kingdom; 3 Department of Mathematics and Computer Science, University of Southern Denmark, Odense, Denmark; 4 Max-Planck Odense Center on the Biodemography of Aging, Odense, Denmark

**Keywords:** Sex ratio at birth, Fertility decline, Son preference, Sex-selective abortion, Microsimulation

## Abstract

We present a micro-founded simulation model that formalizes the “ready, willing, and able” framework, originally used to explain historical fertility decline, to the practice of prenatal sex selection. The model generates sex ratio at birth (SRB) distortions from the bottom up and attempts to quantify plausible levels, trends, and interactions of son preference, technology diffusion, and fertility decline that underpin SRB trajectories at the macro level. Calibrating our model for South Korea, we show how even as the proportion with a preference for sons was declining, SRB distortions emerged due to rapid diffusion of prenatal sex determination technology combined with small but growing propensities to abort at low birth parities. Simulations reveal that relatively low levels of son preference (about 20 % to 30 % wanting one son) can result in skewed SRB levels if technology diffuses early and steadily, and if fertility falls rapidly to encourage sex-selective abortion at low parities. Model sensitivity analysis highlights how the shape of sex ratio trajectories is particularly sensitive to the timing and speed of prenatal sex-determination technology diffusion. The maximum SRB levels reached in a population are influenced by how the readiness to abort rises as a function of the fertility decline.

**Electronic supplementary material:**

The online version of this article (doi:10.1007/s13524-016-0500-z) contains supplementary material, which is available to authorized users.

## Introduction

Since the 1980s and 1990s, several countries in Asia and the Caucasus have witnessed a rise in the proportion of male births compared with female births, conventionally expressed in terms of the sex ratio at birth (SRB) (Guilmoto 2009, 2015). The rise in SRBs within a short span of time, usually a decade or less, make this trend unprecedented, and the sizable populations where it has been noted—for example, in China, India, South Korea, Vietnam, Georgia, and Azerbaijan—make it one of the “most notable anomalies” of contemporary demography (Guilmoto 2009:519). Earlier literature presumed that widespread son preference would keep fertility levels higher than they would be in its absence and thus would delay the fertility transition (Amin and Mariam 1987; Nath and Land 1994). In recent decades, however, SRBs have risen because prenatal sex determination technology that enables couples to resort to sex-selective abortion has spread in contexts where couples desire at least one son as well as a small family size.^1^ This trend has led demographers to speculate that SRBs may rise in other contexts where son preference persists but where safe, effective, and inexpensive sex determination technology has yet to become available (Bongaarts 2013).

The causes, patterns, and demographic implications of imbalanced SRBs remain the subject of a large and growing body of research. Extensive demographic research has highlighted SRB levels and trends in a comparative perspective (Attané and Guilmoto 2007; Duthé et al. 2012; Guilmoto 2009, 2012). In contrast, relatively less attention has been paid to understanding the levels and trends of micro-level dynamics underlying SRB trajectories. Given this lacuna in the literature, Guilmoto (2009) presented a framework for the three micro-level preconditions—persistent son preference, declining total family size, and the spread of prenatal sex determination technology—leading to the practice of prenatal sex selection. In the same study, Guilmoto also developed a framework for understanding macro-level SRB trajectories in terms of an “archetypal transition cycle” involving a rise, a leveling off, and eventual decline toward normalcy that he termed the “sex ratio transition.” Bongaarts (2013) developed a similar macro-level framework that relates different stages of macro-level transition patterns of rising, leveling off, and declining SRBs to different stages of the fertility decline.

Although Guilmoto lucidly described the three preconditions that cause SRB imbalances, he acknowledged that existing data preclude a “more detailed decomposition . . . of these distinct dimensions” in explaining observed SRB levels and trends (Guilmoto 2009:535). The literature has not attempted to quantify, for example, what levels and rates of change in son preference over time, rates of technological diffusion, and probabilities of sex-selective abortion plausibly underpin observed SRB and fertility trajectories. Furthermore, the impact of sex-selective abortion on reducing fertility as distinct from a population that exclusively practices differential stopping behavior (DSB)^2^ has not been explicitly addressed.^3^

We present a model that simulates individual-level reproductive behaviors from the bottom up to examine emergent population-level SRB trajectories. In our model, individuals who desire a son practice DSB. Although these individuals wish to control their total fertility levels over time, their son preference does not always allow them to stop childbearing at low parities. Prenatal sex determination technology then emerges as an exogenous stimulus that diffuses steadily, enabling growing proportions of individuals to reconcile their son preference with their aspirations for smaller families. The proportion of individuals desiring sons, their total fertility levels, and those with access to technology all change over time at differing rates, and their interactions produce aggregate SRB trajectories.

We first develop a general model that allows us to describe and formalize the micro-level processes that generate SRB distortions. We adapt the general model to enable calibration with United Nations World Population Prospects (UN WPP) data on mortality, fertility, and population structure (United Nations 2013). We then calibrate the model for South Korea, where distorted SRBs emerged in the 1980s. By the 1990s, SRBs started to level off; and by the mid-1990s, SRBs showed a remarkable turnaround toward normalization. By the mid-2000s, SRBs had already returned to near-normal levels. As the only country that has been through all three stages of the archetypal “sex ratio transition,” calibrating the model to the South Korean case can shed light on the levels and rates of change in son preference, diffusion of technology, and probabilities of sex-selective abortion that plausibly underpinned different stages of the transition.

The calibration shows how even as proportions of the population who felt it necessary to bear a son declined in South Korea from approximately 45 % in the mid-1980s to approximately 25 % in the mid-1990s, South Korean SRBs became distorted. SRBs peaked in the 1990s, when the proportion with son preference in the South Korean population was approximately 30 %. The SRB distortion was likely caused by an early and steady increase in access to prenatal sex determination technology over the 1980s, combined with growing propensities to sex-selectively abort at low parities as fertility declined. The model calibration suggests that the shape of SRB trajectories in South Korea is very sensitive to the timing of the onset of technology as well as the rate of technology diffusion. Peak levels reached by SRB trajectories are very sensitive to increases in the readiness to abort at low parities triggered by declining fertility. Simulations suggest that the impact of sex-selective abortion on fertility levels in South Korea was relatively small. Sex-selective abortion likely reduced fertility levels by 2.5 % to 3.5 % at the end of the 1980s and early 1990s as SRBs peaked. For 1990, simulations suggest that in the absence of sex-selective abortion the TFR would have been approximately 1.65 instead of 1.60.

## Theoretical Background

### Micro-Dynamics and SRB Distortions

From a theoretical standpoint, the steep rise in SRBs and child sex ratios across Asia and the Caucasus despite rapid modernization and development posed a severe challenge to demographic and social theory (Chung and Das Gupta 2007a; Croll 2000). The same modernization forces that made women educated and economically productive and ensured other forms of social insurance for parents were implicitly assumed to erode the norms of son preference as well. Parents would no longer want children for extra labor, income, or social status (Croll 2000:109)—factors that were thought to underpin son preference in pretransitional societies. Moreover, low fertility would combine with greater industrialization and urbanization to shift families away from the extended patrilineal form—in which sons are imperative for family continuity—toward nuclear forms with more equitable sex roles (Goode 1963). However, contrary to theoretical expectations, even as levels of fertility steadily declined in the 1980s and 1990s across South Korea, China, and India, distorted SRBs seemed to suggest that son preference persisted.

A number of significant studies have noted that indicators of stated son preference, measured in different ways, appeared to decline even as sex ratios worsened (Bhat and Zavier 2003; Bongaarts 2013; Chung and Das Gupta 2007a). In attempting to reconcile these ostensibly paradoxical trends, these studies acknowledged the role of the diffusion of prenatal sex determination technology and fertility decline in contributing to masculine sex ratios amidst declining son preference. These studies, however, did not explicitly model the interplay of these preferences with technology diffusion and fertility decline at the individual-level and link them with macro-level SRB trajectories, nor did they attempt to quantify the micro-level behaviors underpinning macro-level patterns. This study highlights and extends insights on the micro-level underpinnings of SRB trajectories.

### The Sex Ratio Transition

Guilmoto (2009) provided a valuable theoretical framework of the micro-level factors underpinning macro-level SRB distortions. The recent rise in SRBs across Asia, he argued, “resembles a diffusion process similar to that sometimes claimed to be characteristic of a fertility decline” (Guilmoto 2009:524). By likening the shape of the SRB transition to the fertility decline, Guilmoto adapted the well-known “ready, willing, and able” (RWA) approach, which Coale (1973) originally used to account for the European fertility decline in the nineteenth century, to the practice of sex selection. Within this framework, sex-determination technology can be conceptualized as “the key innovation that permits couples to resort to sex-selection in a context characterized by declining fertility and entrenched son preference” (Guilmoto 2009:524).

The practice of sex-selection from the actor or individual’s perspective can be seen as the outcome of three conditions being met: (1) *willingness* to consider sex selection because of the persistence of cultural norms that reinforce the value of male offspring; (2) *ability* to seek sex selection given the availability of relatively affordable and accurate prenatal sex-determination technology and relatively liberal abortion legislation; and (3) *readiness* to practice sex selection as a consequence of the fertility squeeze wherein individuals wish to reconcile their sex preferences with a small total family size. The idea of readiness gains importance as fertility declines with the diffusion of norms toward smaller families wherein prenatal sex selection becomes preferable to additional births as the means to realize son preference.

In the same study, Guilmoto speculated whether South Korean SRB trajectories may be a manifestation of an archetypal transition cycle involving three phases: (1) a rise; (2) a leveling off; and (3) a decline and eventual return to normal levels that may eventually come about across other parts of Asia where similar SRB distortions have been observed. Although the return to normal levels suggestive of a completed transition has been observed only in South Korea, the first (steep rise) and second (leveling off) phases have also been observed in other SRB trajectories, such as those of China and India.

What are the levels and trends of the three micro-level preconditions (willingness, ability, and readiness) that explain distinct phases of the SRB transition? The plausible levels of these three preconditions that inform the shape of the trajectories in these two phases and scenarios for their likely projection into the third phase requires a formalization of each of the three conditions within a flexible, dynamic model. The microsimulation model enables us to simulate the differing rates of change across each of these dimensions at the individual-level to approximate macro-level trajectories.

## Model Description

### Microsimulation and Agent-Based Models

*Individual-based simulation techniques*—a term used to describe both microsimulation and agent-based modeling approaches—have been actively used to model demographic processes, such as kinship structures and kinship resources (Wachter 1997; Zagheni 2011), marriage (Billari et al. 2007; Grow and Van Bavel 2015), and the transition to parenthood (Aparicio Diaz et al. 2011; Winkler-Dworak et al. 2015). Although both approaches take individuals as the unit of analysis and use computer algorithms (Monte Carlo methods) to determine individual transition between states or the adoption of behavioral rules, the different goals and data requirements of the two simulation approaches have often been used to classify them separately (Zagheni 2015).

Microsimulation models rely on empirical transition rates and have been predominantly used for predictive purposes. Agent-based models are concerned with showing the emergence of interesting macro-level patterns from the behavioral rules of individual agents. In contrast to microsimulation approaches, agent-based models often tend to model interaction between agents and the environment, adaptation to stimuli, and other types of social learning or feedback effects. Despite their different classification, Bijak et al. (2013) and Zagheni (2015) have noted that the distinction between the two approaches, at least with respect to demographic applications, is not always clear-cut. Agent-based models in demography often include empirical transition rates to model fertility and mortality, and microsimulation models often include behavioral rules and feedback mechanisms. Our model borrows from both approaches and attempts to show how complex macro-level SRB trajectories can emerge from simple micro-level driving forces, and attempts to indirectly estimate these.

### State Variables

The model^4^ comprises individual agents who each have an identity number (*id*), age (*x*), sex (*s*), cohort (*c*), son preference (*sp*), parity (*p*), sons (*so*), technology access (*tech*), and abortions (*ab*). Table 1 lists agents’ state variables and their values. The model is initialized as a one-sex model with initial female agents; however, as we model male as well as female births, the model becomes two-sex from the first time-step onward.

**Table 1:** Agent’s state variables

Agent’s State Variables	Variable Name	Values
Identity Number	*id*	1, 2, 3, . . .
Age	*x*	0–50
Sex	*s*	1: female
		2: male
Cohort	*c*	Five-year cohort
		(1930–1934, . . . , 2005–2009)
Son Preference	*sp*	0, 1
Parity	*p*	0, 1, 2, . . .
Sons	*so*	0, 1, 2, . . .
Access to Technology	*tech*	1: true
		0: false
Abortions	*ab*	0, 1, 2, . . .

An agent’s identity number, sex, and cohort are assigned to the agent at birth and remain the same throughout the life course. Parity (*p*) corresponds to the current parity of the female agent. Sons (*so*) refer to her number of sons. Access to technology (*tech*) is a Boolean variable that takes a *true* or *false* value depending on whether an agent has access to prenatal sex-determination technology. Abortion (*ab*) indicates how many sex-selective abortions a female agent has over her life course. An agent’s age, parity, son preference, sons, access to technology, and abortion values are time-varying and are updated at each time-step in the model.

### Initialization

To approximate the initial parity distribution of South Korean women from 1980 onward, we initialized the model 35 years earlier in 1945 to allow all women in reproductive ages (15+) to complete their fertility careers by 1980 and have their children belong to the starting population of 1980. Using UN WPP data, we approximate the South Korean population structure of 1945 and start a simulation in which individuals die and reproduce according to their age-specific death and fertility rates for each year (United Nations 2013).^5^ The abortion procedure is not modeled prior to 1980. The resulting population structure obtained in 1980 is very close to the population structure of South Korea reported in UN WPP, and the minor differences that persist are likely attributable to migration dynamics that are not modeled in our initialization procedure.

### Procedures

The model contains two procedures for agents: (1) aging and (2) reproduction, both of which are carried out at each time-step (tick) in the model. Each tick corresponds to one year. At each tick, an agent ages by one year, and time moves forward by one year. The model uses sex- and age-specific mortality rates until age 50 from UN WPP to model aging (United Nations 2013). Because we focus on reproductive behavior, all female and male agents who survive are removed from the simulation at age 50. By simulating male agents until age 50, we can account for child and young adult mortality for males, which may have an impact on a woman’s reproductive behavior, as we describe later. If a woman were to lose her son during childhood, she might then be able to attempt to have a son again.

The reproduction procedure models conception and birth for female agents. The risk of childbirth *h*_*i*_(*x*_*i*_, *t*) for each female agent *i* is determined by the age-specific (*x*_*i*_) fertility rates for that period (*t*) from UN WPP (${h}_i^{*}\left({x}_i,t\right)$), her son preference (*sp*_*i*_), and her current number of sons (*so*_*i*_). The sex of the birth is determined at the point of conception by a probability of 0.5122 for male births and 0.4878 for female births, which corresponds to an SRB of 105.^6^ We model sex-differential birth-stopping behavior; and as our model moves in time and technology becomes available, we model the opportunity for female agents to have sex-selective abortions. Details of how these behaviors are modeled in our agents are described in the next section.

### Modeling Reproductive Behaviors

#### Sex Selection: Ready, Willing, and Able (RWA)

**Fig. 1:**
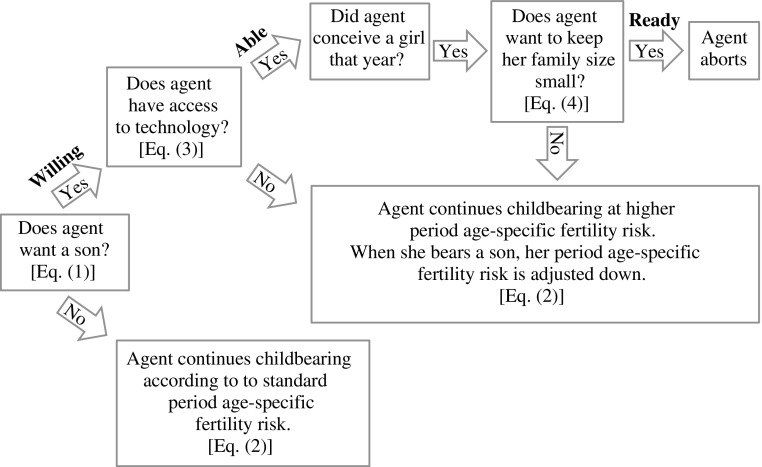
Diagram of each simulation step

The model formalizes the RWA framework to the practice of prenatal sex selection as presented in Guilmoto (2009). A woman’s decision to practice sex selection is the outcome of three processes that are modeled as probabilities, in this order: (1) whether she has a son preference (willing), (2) whether she has access to technology (able), and (3) whether she feels a fertility squeeze to restrict her total family size while realizing her son preference (ready). Figure 1 illustrates how each simulation step proceeds.

#### Son Preference (Willingness)

Son preference (*sp*_*i*_) is assigned as follows: agents have either no preference for male offspring (*sp*_*i*_ = 0) or a desire for one male offspring (*sp*_*i*_ = 1). Only women in their reproductive ages have a son preference. Those who have son preference (*sp*_*i*_ = 1) practice DSB: that is, they have higher fertility rates than those who do not or have met their son preference (see the following section on DSB).

**Fig. 2:**
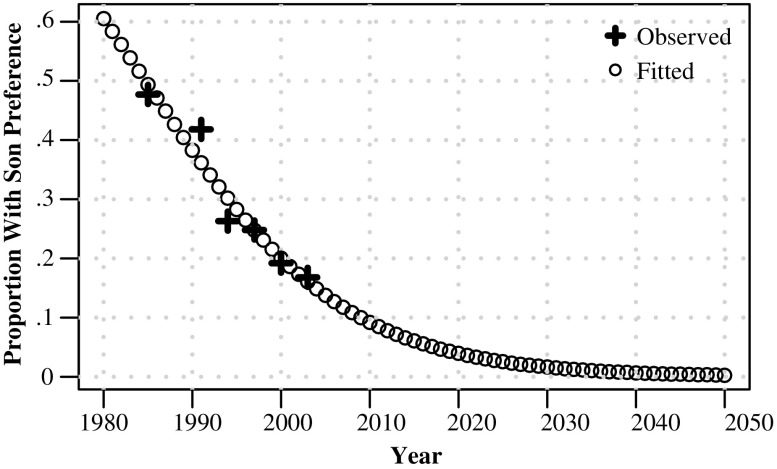
Proportion with son preference: South Korea, 1980–2050. Observed values from Chung and Das Gupta (2007b)

For South Korea, we approximate an individual’s probability of having son preference as time-varying, using data from a question that asks women whether they feel that they must have a son.^7^ We rely on time trends in proportions stating they must have a son, as reported in Chung and Das Gupta (2007b). Given that we are modeling a dichotomous proportion that is bounded between 0 and 1, we fit a logistic regression (Eq. (1)) with one predictor variable (δ_*t*_) and an intercept term (δ_0_) to obtain yearly probabilities of son preference for the period between 1980 and 2050. Figure 2 shows the observed and fitted son preference trends from Eq. (1).^8^

1 $$ sp(t)=\frac{e^{\updelta_0 + {\updelta}_t}}{1+{e}^{\updelta_0 + {\updelta}_t}}. $$

We choose to assign son preference dichotomously because we believe this effectively captures the way individuals experience son preference, by finding it imperative to bear a son. This measure also allows for easy interpretation for how much son preference exists in a population. Although some individuals—particularly among older cohorts—may desire more than one son, the desire for several sons likely reflects the indirect influence of higher mortality conditions where bearing at least two sons might be considered a strategy to ensure at least one survived into adulthood. By simulating mortality dynamics for males until age 50, this feedback effect of high mortality levels on fertility behavior is accounted for in the model as mentioned in the earlier section on procedures.

#### Differential Stopping Behavior

Differential stopping behavior (DSB) is a common manifestation of son preference (Arnold et al. 1998; Clark 2000; Larsen et al. 1998; Retherford and Roy 2003). In the model, female agents with unmet son preference have a higher fertility risk, expressed as a deviation from the standard fertility schedule ${h}_i^{*}\left({x}_i,t\right)$ by a proportional expansion factor (1 + γ).

2 $$ {h}_i\left({x}_i,t\right)=\left\{\begin{array}{ll}{h}^{*}\left({x}_i,t\right)\hfill & \mathrm{if}\ s{p}_i(t)=0,\hfill \\ {}{h}^{*}\left({x}_i,t\right)\times \left(1+\upgamma \right)\hfill & \mathrm{if}\ s{o}_i(t)<s{p}_i(t),\hfill \\ {}{h}^{*}\left({x}_i,t\right)\times \left(1 - \upalpha \right)\hfill & \mathrm{if}\ s{o}_i(t)\ge s{p}_i(t)\ \mathrm{and}\ s{p}_i(t)\ne 0.\hfill \end{array}\right. $$

As Eq. (2) shows, if the current number of sons *so*_*i*_(*t*) of female agent *i* is less than her son preference *sp*_*i*_(*t*), her period age-specific rate is multiplied by a factor of (1 + γ), where γ may be conceptualized as a son preference–intensity parameter. For example, γ = 0*.*2 implies that a woman with unmet son preference experiences a fertility risk that is 20 % higher than the period age-specific schedule that normally determines her risk for childbearing. A higher value of γ indicates a higher intensity of son preference through its impact on fertility behavior.

When son-preferring female agents bear a son, their birth risk is adjusted down by a factor of (1 − α), indicating a reduced risk from the standard fertility schedule ${h}_i^{*}\left({x}_i,t\right)$. Standard period age-specific rates *h* ^*^(*x*_*i*_, *t*) apply to female agents with no son preference (*sp*_*i*_ (*t*) = 0).

#### Access to Technology (Ability)

We use the logistic diffusion model, widely used to describe the diffusion of new technologies, to model an individual’s ability or probability of gaining access to technology (Geroski 2000).

3 $$ Ability(t)=\frac{e^{\uprho \left(t - \upphi \right)}}{1+{e}^{\uprho \left(t - \upphi \right)}}. $$

In Eq. (3), *Ability*(*t*) simulates an individual’s probability of getting access to technology, which increases as a function of time (*t*), where *t* corresponds to the time-step in the simulation (*t* = 0, 1, 2, . . . , 30 for a 30-year simulation covering the period 1980–2010), ρ determines the slope or rate of increase, and ϕ is the inflection point of the logistic diffusion curve. At the population level, *Ability*(*t*) can be interpreted as the proportion of individuals at a particular time-step gaining access to prenatal sex-determination technology. At each time-step, *Ability*(*t*) is recalculated, and a random number from a uniform distribution for each individual is redrawn, which when less than *Ability*(*t*) sets the state variable *tech*_*i*_(*t*) = true for that individual.

#### Fertility Decline (Readiness)

As fertility falls, norms surrounding smaller families become more entrenched. Individuals are likely to desire smaller families; and if means are available to allow them to realize their son preference with small family size, they are likely to do so. This is the motivating idea to generate an individual’s probability (readiness) to abort. Guilmoto (2009) described the readiness to abort as strongly related to the fertility squeeze felt by couples planning the size and composition of their families. From a modeling perspective, this fertility squeeze can be viewed as a form of social pressure that is closely related to prevailing total fertility levels and determines an individual’s readiness to abort. This readiness to abort is likely higher when fertility levels are lower, couples feel a greater squeeze or pressure to reconcile their son preference at lower parities than when average family size is higher, and proceeding to higher parities is not out of step with prevailing total fertility norms.

Equation (4) shows how we model readiness to sex-selectively abort. A woman is ready only if she has unmet son preference (willing) and she has access to technology (able): that is, if *sp*_*i*_(*t*) > *so*_*i*_(*t*), and *tech*_*i*_(*t*) = true. Hence, if these two conditions are met,

4 $$ Readines{s}_i(t)=\left\{\begin{array}{cc}\hfill \min \left\{1,\frac{\upbeta}{\mathrm{TFR}\left(t - 1\right)}\right\}\hfill & \hfill \mathrm{if}\ {p}_i(t)=0,\hfill \\ {}\hfill \min \left\{1,\frac{p_i(t)\times \upsigma}{\mathrm{TFR}\left(t - 1\right)}\right\}\hfill & \hfill \mathrm{if}\ {p}_i(t)>0,\hfill \end{array}\right. $$

where *p*_*i*_(*t*) denotes the parity of agent *i* at time *t*. Because *Readiness*_*i*_(*t*) is a probability to abort, it is bounded between 0 and 1.

An agent’s readiness to abort depends on her (1) current parity (*p*_*i*_(*t*)) at the beginning of the period; (2) prevailing, model-generated^9^ fertility levels, TFR(*t* − 1); and (3) two parameters σ (if *p*_*i*_(*t*) > 0) and β (if *p*_*i*_(*t*) = 0).^10^ In Eq. (4), the ratio of the agent’s current parity and prevailing fertility levels is conceptualized as determining the extent of her fertility squeeze. When fertility levels are higher—for example, at a TFR of 3 children per woman—a woman with unmet son preference and access to technology has a 0*.*33 × σ probability to abort as she transitions from first to second parity compared with a woman who transitions from first to second parity when total fertility levels have fallen to 2*.*5 and the probability is 0*.*4 × σ. The parameter σ allows us to assess the impact of the fertility squeeze by scaling it up or down on SRB trajectories when calibrating the model. It also allows us to account for the possibility that even if the fertility squeeze may be present in a population, there might be other counteracting forces—such as religious or cultural taboos against the practice of abortion, or punitive measures against sex-selective abortion—that may not allow for the full extent of the fertility squeeze to be felt. Conversely, higher σ values indicate a greater intensity of the fertility squeeze.

Indeed, in some situations, particularly as fertility becomes very low, we may expect some women to abort at the lowest possible parity: that is, parity 0 or before the transition to first birth. Although abortions before the first birth may become more frequent as fertility falls, these events tend to be more rare than higher-parity abortions (Guilmoto 2009:533). We therefore model parity 0 abortions as a function of prevailing fertility levels but subject to their own parameter β than higher-parity abortions, which are controlled by the parameter σ. Table 2 lists the relevant parameters that control the three factors in the model: son preference, fertility decline, and technology availability. The range listed in the table refers to theoretically plausible values for each parameter over which we analyze model behavior.

**Table 2:** Simulation model parameters

Category	Parameter	Range	Description
Son Preference (willingness)	γ	[0, 1]	Son preference intensity
	α	[0, 1]	Birth risk adjustment (when son preference realized)
Fertility Decline (readiness)	σ	[0, 2.5]	Fertility squeeze scaling factor for parity 1 and higher
	β	[0, 0.5]	Fertility squeeze scaling factor for parity 0
Access to Technology (ability)	ρ	[0, 2]	Rate of technology diffusion
	ϕ	[0, 20]	Inflection point for technology availability

## Simulation Results

### Model Calibration for South Korea

We calibrate the model for South Korea by seeking to match the shape and levels of model-generated SRB trajectories with UN WPP estimates of the same for the period, 1980–2010 (United Nations 2013). We find the combination of parameters that minimize the average yearly error (model fit measure, or root mean squared error (RMSE)) between the simulated SRB trajectories and UN estimates of South Korean SRBs. The model fit measure can be expressed as follows:

5 $$ \mathrm{Model}\kern0.5em \mathrm{Fit}\kern0.5em \mathrm{or}\kern0.5em \mathrm{R}\mathrm{MSE}=\sqrt{\frac{{\displaystyle \sum {\left(UN\ SRB(t) - \mathrm{S}\mathrm{imulated}\ \mathrm{S}\mathrm{R}\mathrm{B}(t)\right)}^2}}{2010 - 1980}.} $$

Low values of the RMSE model fit measure indicate better fit with smaller average yearly errors between model-generated SRBs and UN SRB estimates. We also attempt to calibrate model-generated TFR levels to UN WPP estimates of the same because the model-generated TFR forms an important part of the readiness condition, as shown in Eq. (4).

**Fig. 3:**
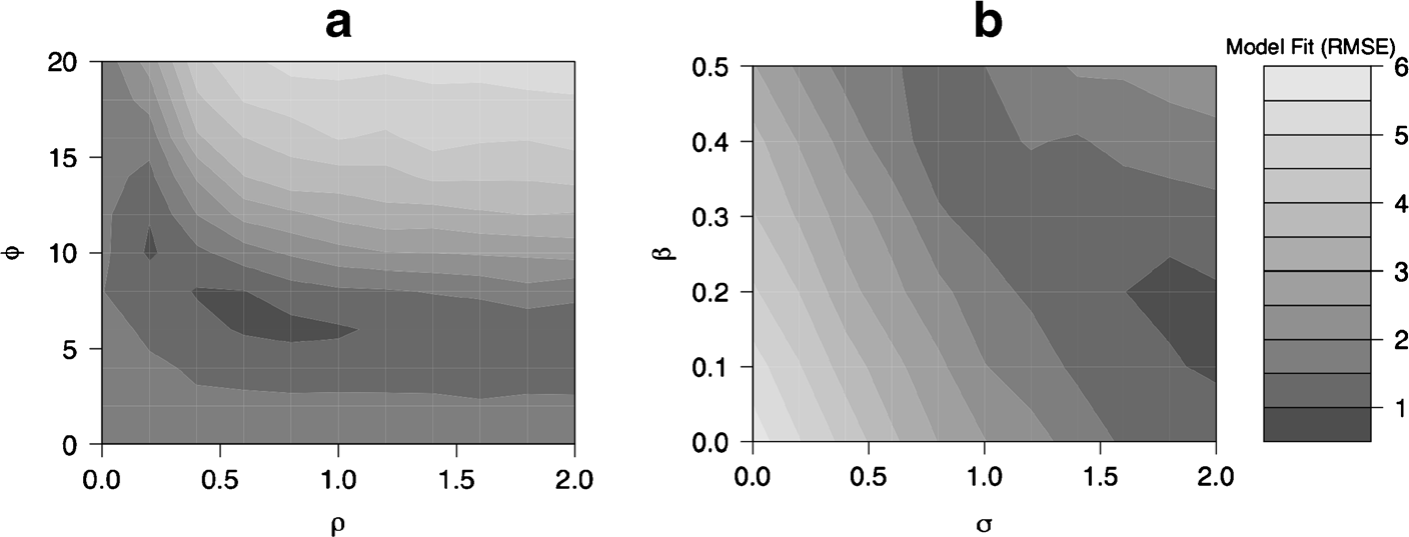
Model fit measure or root mean squared error (Eq. (5)) in response to varying ability parameters (ρ and ϕ in panel a), and varying readiness parameters (σ and β in panel b). Lower values indicate better fit between simulated and UN SRB estimates

Our calibration strategy proceeds as follows: we run the model on an exploratory Latin hypercube sample comprising 224 design points. One simulation was run at 180 of the 224 design points, and five repetitions were performed at 44 randomly chosen points in the sample, totaling 400 simulation runs ((180 *×* 1) + (44 × 5)).^11^ Each design point corresponds to a unique combination of the six model parameters. These design points range over the minimum and maximum values for the parameters listed in Table 2. From this sample of points, we interpolate across all possible combinations of the parameter space (total of 548,856 design points) using a regression metamodel, and explore the sensitivity of the model to different regions of the parameter space that give us good fit (for more details, see the upcoming section Sensitivity Analysis and Online Resource 1). Regions where the simulated SRB trajectories match UN estimates for South Korea well (with low model fit or RMSE values) indicate early (low ϕ) and steady technology diffusion (ρ values between 0.5 to 0.6) as well as a strong readiness to abort (σ values upward of 1.7 combined with smaller values of β in the region of 0.2). SRB fit is expectedly less sensitive to α and γ. We run the simulation at a combination of levels varying the ability and readiness parameters, as shown in Fig. 3. In panel a of the figure, we vary ability parameters ρ and ϕ across their range while holding σ = 1*.*7 and β = 0*.*2; and in panel b, we vary readiness parameters σ and β while holding ρ = 0*.*5 and ϕ = 7.

Data on son preference from South Korean fertility surveys indicate that stated son preference declined from 48 % in 1985 to 26 % in 1994; over the same time, South Korean SRBs rose from around 108 to 114, and fertility declined around 2.1 to 1.6. It is interesting to note that son preference levels appear to be relatively low (approximately 30 % of the population stating “must have son”) as SRBs reached their peak in 1990.

**Fig. 4:**
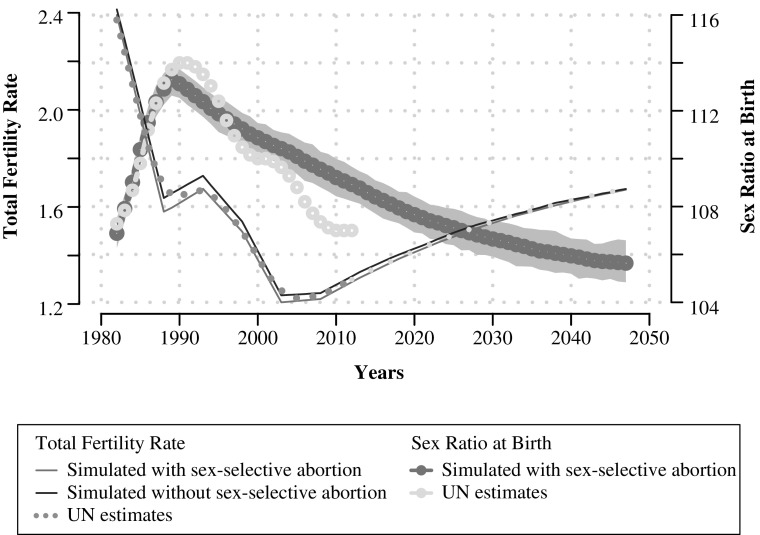
Simulated total fertility rate (TFR) and sex ratio at birth (SRB) trajectories, five-year moving averages: South Korea, 1980–2050. Gray band indicates 95 % empirical confidence interval of simulated SRB trajectories

Figure 4 shows model-generated SRB and fertility trajectories with 95 % empirical confidence interval bands that best match UN WPP SRB trajectories, which are shown in the background in gray.^12^

**Fig. 5:**
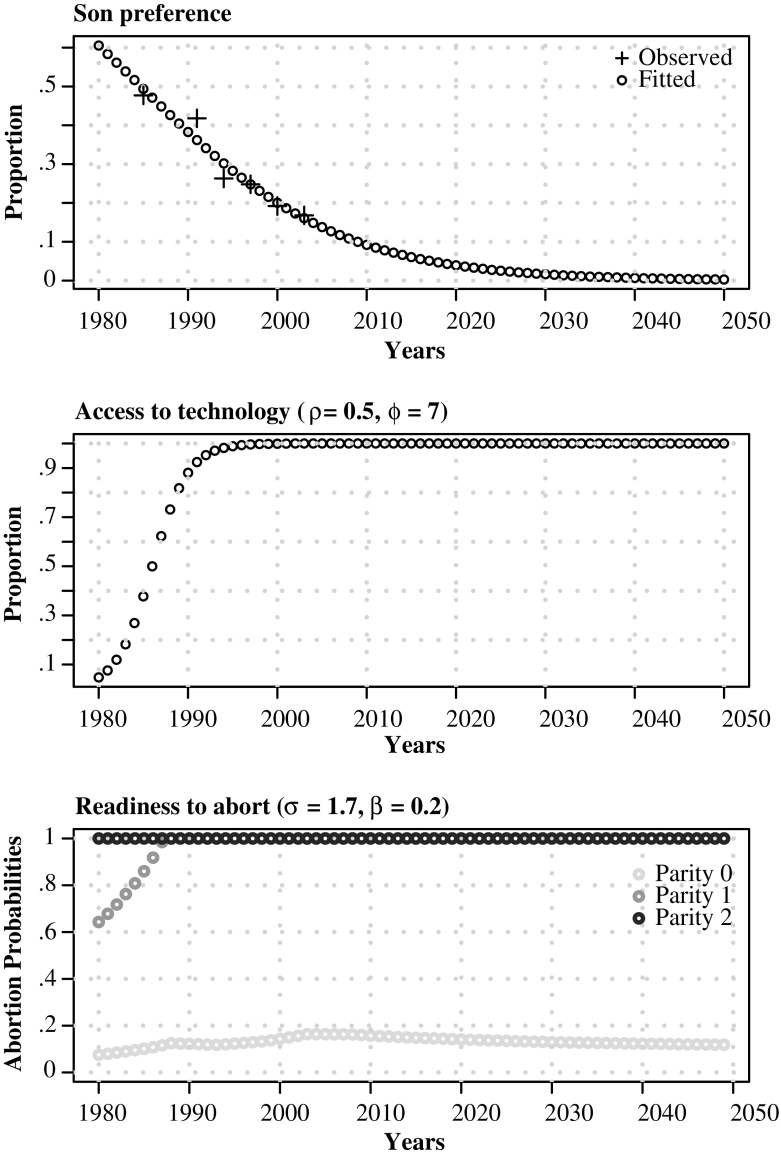
Son preference (willingness), access to technology (ability), and parity-specific abortion probabilities (readiness) underlying calibrated model: South Korea, 1980–2050

The dotted-dashed SRB curve in Fig. 4 shows five-year moving averages of SRBs averaged across 240 simulation runs with an initial population of 1,000,000 individuals, with the following parameter values: γ = 0*.*20*,* α = 0*.*075*,* ρ = 0*.*5*,* ϕ = 7*,* σ = 1*.*7, and β = 0*.*2. Increasing β to levels closer to 0.25 allows us to match the peak better but at the expense of worsening the fit for the period 1998 onward, thereby worsening the average yearly error between the simulated and UN SRB estimates. Our simulated fit, while capturing the general shape and SRB levels well, does not capture the drastic turnaround toward normalization that occurred in the mid-2000s. The dynamic trajectories and associated parameters of willingness (son preference), ability (technology access), and readiness (abortion probabilities as a function of the fertility squeeze) that underlie the simulated SRB curve reported in Fig. 4 are shown in Fig. 5. The technological diffusion trajectories indicate saturation by the mid-1990s, with the steepest rise in the 1980s, combined with an increase in the probabilities to abort in the mid-1980s that lead to the rise in the SRB, even as son preference continued to decline throughout the period.

A son preference intensity parameter of γ = 0*.*20 and birth risk adjustment of α = 0*.*075 allow us to match Korean fertility trajectories over the period with the prevailing levels of son preference very well. How much did the practice of sex-selective abortion hasten the fertility decline in South Korea? To answer this question, we adopted a counterfactual strategy assuming that no sex-selective abortion was practiced (σ = 0 and β = 0), keeping all other parameters constant at the levels used to generate the SRB distortions. The solid black line shows TFR levels assuming that no sex-selective abortion had been practiced. When SRBs peaked at the end of the 1980s and early 1990s, the TFR levels in the no sex-selective abortion scenario are about 2.5 % to 3.5 % higher than with sex-selective abortion. The difference in TFR levels between the two scenarios is greatest between the years 1990 and 1995 when it was at 0.05, which can be interpreted as 5 births per 100 mothers of childbearing age. For 1990 when SRBs peaked, in the absence of sex-selective abortion, simulations suggest that the TFR would have been approximately 1.65 instead of 1.60.

### Experimental Scenarios

**Fig. 6:**
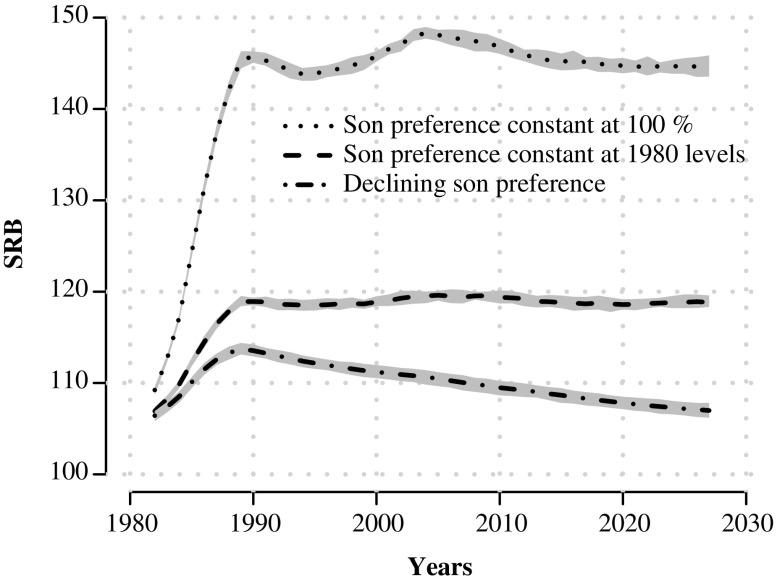
Simulated sex ratio at birth (SRB) trajectories, five-year moving averages, over 50 years, assuming son preference remains constant at 1980 levels (50 %) and at 100 %. Comparison with simulated SRB trajectories shown in Fig. 4. Gray band indicates 90 % confidence interval

We present two sets of counterfactual scenarios to show how SRB trajectories respond to different assumptions about willingness, readiness, and ability. First, we compare how SRB trajectories would have looked had son preference (willingness) been constant (1) at 1980 levels of 50 %, and (2) at 100 %. We keep technology diffusion early and steady at the same parameters as in Fig. 4 (ϕ = 7 and ρ = 0*.*5) along with strong readiness parameters of σ = 1*.*7 and β = 0*.*2. We compare these against the simulated SRB trajectories presented in Fig. 4. All three SRB trajectories are shown in Fig. 6.^13^

In both constant scenarios, SRB trajectories stabilize at high levels by the early 1990s and do not show a turnaround. If son preference is kept at 1980 levels, SRBs stabilize at levels of 120; and in the 100 % scenario, they stabilize at very high levels of 145—levels that have not been observed in national-level SRB trajectories anywhere.

**Fig. 7:**
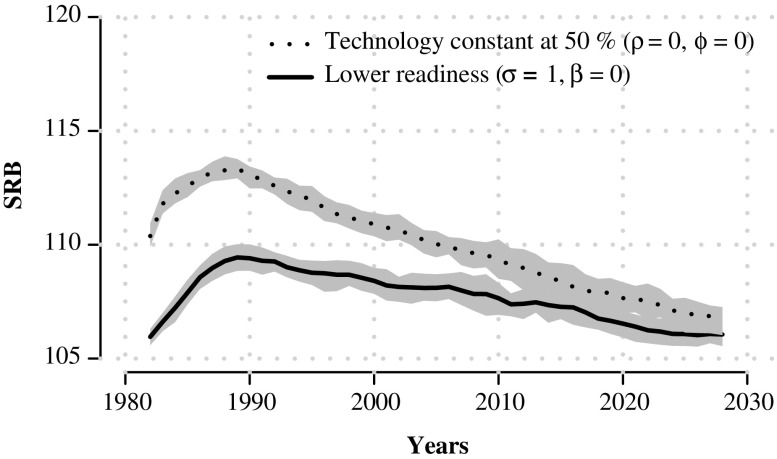
Simulated sex ratio at birth (SRB) trajectories, five-year moving averages, over 50 years assuming that (1) technology availability stays constant at 50 % throughout the simulation period, and (2) fertility squeeze is less intense with readiness parameters σ = 1 and β = 0. Gray band indicates 90 % confidence interval

In the second set of scenarios, we modify assumptions about ability and readiness. These are shown in Fig. 7, which assumes that (1) technology availability stays constant at 50 % throughout the simulation (ρ = 0 and ϕ = 0, and σ = 1*.*7 and β = 0*.*2); and (2) that the fertility squeeze felt by individuals is less intense, leading to a lower readiness to abort with no parity 0 abortion (σ is reduced to 1 instead of 1.7 in Fig. 4; and β is reduced to 0 instead of 0.2, with ρ = 0*.*5 and ϕ = 7). In Scenario 1, the rise in SRB levels is triggered by increasing readiness to abort induced by the fertility squeeze, but SRB levels start high in 1980 and don’t capture the shape between 1980 and 1990 well. In Scenario 2, SRB levels remain much lower, reaching a peak of 110 instead of 113–114 when σ and β are higher in the best-fit simulated trajectories.

### Sensitivity Analysis

How much of the variance of model outcomes is accounted for by different parameters? We address this issue by sensitivity analysis of three model outcomes: (1) the model fit or RMSE measure, which is the average yearly error for 1980–2010 between simulated and UN estimates of South Korean SRB trajectories (see Eq. (5)); (2) the South Korean SRB for 1990, when the SRB peaks according to UN estimates; and (3) the maximum SRB level reached by the model. We estimate regression metamodels on data generated by the simulation model.^14^ These models separately take each of the three outcomes of interest as the dependent variable, with the parameter values as the predictor variables in the regression model. Regression metamodeling for simulation models is widely used in the sensitivity analysis of simulation models (Kleijnen 1995; Santner et al. 2003) and has been used in agent-based modeling applications in demography (Grow and Van Bavel 2015). After finding a well-fitted metamodel for each outcome, we perform an analysis of variance (ANOVA) of the regression metamodel to assess how much of the model variance is accounted for by each parameter (Santner et al. 2003: chapter 7). The variance explained by each parameter as well as selected, statistically significant (*p < .*05) higher-order terms and interactions from the ANOVA decompositions are reported in Table 3. Further sensitivity analysis results are reported in Online Resource 1.

**Table 3:** Proportion of variance (%) of three model outcomes: (1) model fit measure, (2) SRB 1990, (3) maximum SRB attributable to model parameters and their interactions

(1) Model Fit Measure		(2) SRB 1990		(3) Maximum SRB	
Parameter	Variance	Parameter	Variance	Parameter	Variance
α	1.54	α	0.34	α	0.00
γ	0.50	γ	3.09	γ	14.30
σ	0.30	σ	9.75	σ	28.28
β	0.40	β	12.93	β	20.24
ϕ	26.40	ϕ	29.70	ϕ	4.93
ρ	2.50	ρ	5.53	ρ	0.50
Higher-Order Terms		Higher-Order Terms		Higher-Order Terms	
ρ^2^	5.40	ρ^2^	9.14	ρ^2^	1.65
σ^2^	2.60	σ^2^	3.23		
γ^2^	1.11	γ^2^	1.45		
Significant Interactions		Significant Interactions		Significant Interactions	
ϕ × ρ	14.50	ϕ × ρ	2.05	σ × ϕ	1.17
σ × ϕ	2.30	σ × ϕ	2.65	β × ϕ	0.67
β × ρ^2^	2.30	γ × ϕ	1.45	γ × σ	0.90
σ × β	8.00	σ × ϕ × ρ	1.67		
γ × σ	4.50				
γ × β	2.35				
Residuals	22.06	Residuals	12.98	Residuals	20.89

*Note:* Variance measures are derived from analysis of variance (ANOVA) of regression metamodels.

Nearly one-half of the variance (44.3 %) in the model fit or RMSE measure can be accounted for by the inflection point for the onset of technology diffusion (ϕ), the quadratic term involving the speed of technology diffusion (ρ), and the interaction of the two technology diffusion parameters. This finding indicates that the shape of the South Korean SRB trajectory, including the rise and turnaround, is especially sensitive to the ability parameters. The interaction terms involving the readiness parameters (σ and β) account for 8 % of the variance, and interaction terms involving γ (the son preference intensity parameter), account for about 7 % of the variance of the model fit measure. Technology diffusion parameters also account for the greatest fraction of the variation in the levels of the South Korean SRB in 1990 (about 47 %, including first-order, second-order, and interaction terms), followed by the readiness parameters of β and σ, which account for about 26 %. The maximum SRB levels reached in the model is most sensitive to the readiness parameters (σ and β), which together account for nearly 50 % of the variation in this outcome. The son preference–intensity parameter γ also accounts for a sizable proportion of the variation in the maximum SRB outcome, although it is more important in its role in interactions with other parameters for the other two outcomes. This result is plausible given that α and γ do not have direct effects on the SRB but effects that are mediated through the TFR in the model.

The regression metamodels incorporating linear, quadratic, and interaction terms for each of the three outcomes capture a sufficiently high fraction of the variance in the output. For the model fit measure, the best-fit metamodel captures 78 % of the model variance (residual variance 22.06 %). The best-fit regression metamodel with the maximum SRB reached in the model as the outcome captures a similar proportion of the variance (79.11 %; residual variance 20.89 %), whereas the metamodel with the South Korean SRB in 1990 captures 87.02 % of the variance (residual variance 12.98 %).^15^

## Discussion

What balance of micro-level behaviors underlie different stages of the sex ratio at birth (SRB) transition at the macro level? What will be the future course of SRB trajectories? By formalizing a general framework for the decision to practice prenatal sex selection—ready, willing, and able—our goal in this study has been to present a model that can be fitted at the individual level to approximate how changes in son preference (willingness), technology diffusion (ability), and the fertility squeeze triggered by the fertility decline (readiness) generate macro-level SRB trajectories.

The model reveals a number of interesting insights on the microdynamics that underpin the sex ratio transition. Even low levels of son preference can cause significant SRB distortions when technology diffuses steadily and fertility falls quickly. The model suggests that the shape of South Korean SRB trajectories between 1980 and 2010—including the steep rise and turnaround—was particularly sensitive to the inflection point for technology diffusion (ϕ), followed by the rate of technology diffusion (ρ). The maximum SRB levels were highly sensitive to the readiness to abort, controlled by σ and β, which jointly accounted for nearly 50 % of the variation in the maximum level reached. As SRB levels steadily rose in the late-1980s to the early-1990s in South Korea, we estimate that sex-selective abortion likely reduced fertility levels slightly, between 2.5 % and 3.5 %.

The simulation model presented here provides a sufficiently general and useful approach that can be adapted to different contexts. The experimental scenarios demonstrate that the model is capable of generating SRB trajectories of different peak levels and shapes from different parameter combinations of the three underlying processes. In other contexts, such as China (where SRBs have peaked at levels higher than in South Korea), we would likely need higher values of the readiness parameters σ and β than in South Korea because of the additional fertility squeeze triggered by the one child policy, combined with a slightly later and slower technology diffusion than that in South Korea, which is a richer country. On the other hand, we showed in a separate study (Kashyap and Villavicencio, 2016) the plausibility of slower technology diffusion parameters combined with a lower readiness to abort in the higher fertility context of India, where SRB trajectories have been flatter and less peaked than South Korea. In future work, we plan to explore which parameters are relevant for different SRB trajectories.

It is important to acknowledge some limitations of the model, especially when considering it as a tool for projecting SRB trends. We have sought to develop the model to enable calibration with widely available UN data on mortality, fertility, and age structure. However, data on fertility preferences—particularly information on son preference similar to that found in Korean fertility surveys—may be harder to come by or may need to be indirectly estimated from other questions. Other, more extensive parameterizations for son preference that borrow from best-available data sources would be useful extensions for the model for projection purposes and for wider applicability.

We do not explicitly account for regional heterogeneity in the model in its current stage. We account for heterogeneity in the micro-level processes driving the macro-outcome in the model over time, and regional heterogeneity may be seen as one implicit component of this overall heterogeneity. Nevertheless, an aggregate picture at the national level may be composed of extremely disparate trends at the regional level, whose impact cannot be explored presently.

Beyond the individual-level mortality feedback effects on son preference for an individual, we also do not model other social feedback mechanisms and social network effects that may have important implications for understanding how son preference changes over time in a population. Indeed, this may be a reason why our simulation does not fully capture the dramatic turnaround in the 1990s in the South Korean SRB trajectory. The approximations of the three underlying processes for South Korea that our model generates come close to—albeit not close enough—explaining the significant change in the SRB that was observed in the country. More work on the exact mechanisms of change in son preference is certainly needed. We have chosen to refrain from incorporating such mechanisms at present because we believe that the research on mechanisms driving the shift in son preference norms is nascent. Qualitative studies focused on understanding mechanisms underpinning changes in norms could provide valuable insights that can be incorporated in simulation approaches to examine their macro-level implications. In extensions and applications of the model, we hope to incorporate these as well as address the other issues that we have raised. We also invite other researchers to extend and improve the model.

## Electronic supplementary material

ESM 1 (DOCX 41 kb)

ESM 2 (ZIP 788 KB)

## Footnotes

1 Sex-selective abortion requires prenatal sex-determination technologies as well as methods for abortion. The currently available technologies for prenatal sex-determination are (1) *amniocentesis*, an invasive procedure that is conducted between 15 and 20 weeks of pregnancy; (2) *ultrasonography*, which can determine the sex of the fetus as early as 11 weeks; and (3) *blood tests* involving the analysis of the fetal DNA floating in the mother’s bloodstream, which are minimally invasive procedures that can be done at home as early as seven weeks into the pregnancy (Bongaarts 2013).

2 Differential stopping behavior (DSB) refers to fertility behavior in which couples continue childbearing until they reach a desired number of sons by regulating their contraceptive use based on the sex composition of existing children. Several studies have reported the presence of DSB—manifested in higher levels of contraceptive use after bearing sons, higher levels of parity progression in daughter-only families, or high sex ratios at last birth—across different son-preferring populations (Amin and Mariam 1987; Arnold et al. 1998; Bongaarts 2013; Clark 2000; Larsen et al. 1998; Retherford and Roy 2003).

3 An exception here is Lin et al. (2014) who found that the legalization of abortion decreased overall fertility marginally in Taiwan by about 0.01 or 1 birth per 100 women of childbearing age, but increased it for older mothers. Taiwan is a unique context as ultrasound technology was already available before abortion was legalized.

4 The model is programmed in R (R Core Team 2015), and the code is available in Online Resource 2.

5 The UN WPP data are available only from 1950 onward. Consequently, we approximated the population structure of 1945 with the one from 1950; and for the period 1945–1950, we used the same age-specific fertility rates and death rates as for the period 1950–1955.

6 Recent work by Orzack et al. (2015) has found that the natural conception probability is 0.5, but higher female fetal mortality results in the masculine SRB. Given that we do not explicitly model pregnancies, we model the risk of conception to correspond to the SRB.

7 This question has been asked in the Korean fertility surveys carried out by the Korean Institute for Health and Social Affairs.

8 The model is programmed so as to allow an individual’s probability to be son-preferring (*sp* = 1) to be determined by the period (*t*) as well as cohort (*c*) to which she belongs. If cohort-disaggregated data are available, an additional coefficient capturing a cohort effect would need to be estimated in Eq. (1). We were unable to access the survey microdata to acquire cohort-disaggregated data for South Korea. Thus, whether a woman in her reproductive age has a son preference in a particular year is determined by the period probability, which is assumed to be constant across cohorts. Chung and Das Gupta (2007b) noted that period effects were stronger than cohort effects in the decline of stated son preference in South Korea.

9 Although period age-specific fertility rates are an input in the model, they are modified by the son preference intensity (γ) and reduction (α) parameters as well as the proportion of the population with son preference in that year, which results in deviations from the fertility schedule as defined in Eq. (2).

10 Because an agent’s current parity is determined at the beginning of the period *t* and TFR is calculated at the end of the period *t*, the most recently observed TFR with respect to an agent’s parity when she is at risk of childbearing in that period is TFR(*t* − 1).

11 Latin hypercube samples have good space-filling properties, perform better than random samples in assessing model behavior in a comparatively small number of runs, and are recommended good practice in the literature on the design of computer experiments (Santner et al. 2003).

12 UN SRB and fertility trends are issued for five-year periods (1970–1975, 1980–1985, and so on). Our model runs produce yearly estimates. To enable a better comparison between the UN estimates and our simulated results, we present smoothed UN estimates in which we linearly interpolate the five-year values for each year. For this, as advised in UN metadata, we assume that the value for each of the five-year intervals corresponds to midpoint within the interval (e.g., the 1970–1975 rate corresponds to midpoint 1973).

13 In all experimental scenarios, we start with an initial population of 500,000 and repeat the simulation 36 times.

14 As we describe earlier, we use a Latin hypercube sample consisting of 224 design points with five repetitions at 44 of those points, totaling a data set of 400 observations that covers different parameter combinations across the minimum and maximum theoretical values of the parameter ranges.

15 For all three outcomes, higher-order polynomial terms beyond quadratic terms did not account for a statistically significant fraction of the variance and were not included in the models. The residual variance term partly captures simulation stochasticity, along with other variation that cannot be captured by the metamodel.
