# Supplementary material for: The Dynamics of Son Preference, Technology Diffusion, and Fertility Decline Underlying Distorted Sex Ratios at Birth: A Simulation Approach
Source: Demography. 2016 Sep 16;53(5):1261–81. doi: 10.1007/s13524-016-0500-z (PMC5566175; doi:10.1007/s13524-016-0500-z)
Supplement: Supplementary file 1 — (DOCX 41 kb) [file 13524_2016_500_MOESM1_ESM.docx]

**Online Resource 1**

**Sensitivity Analysis**

**The Dynamics of Son Preference, Technology Diffusion, and Fertility Decline Underlying Distorted Sex Ratio at Birth: A Simulation Approach**

**Overview**

The goal of sensitivity analysis with the model is to assess how much of the model variance for three model outcomes can be accounted for by different parameters and their interactions. The three outcomes we are interested in are:

1) the model fit measure (or the root mean squared error, RMSE) that denotes the average error for each year between 1980 and 2010 between the simulated sex ratio at birth (SRB) and UN estimates of SRB. This can be expressed as:

$Model fit= \sqrt{\frac{\sum({UN SRB\left( t \right)-Simulated SRB\left( t \right))}^{2}}{2010-1980}}$ (1)

2) the South Korean SRB in 1990, when the SRB peaks; 3) the maximum SRB level attained by the model. Running the model at all-possible combinations of the six parameters is computationally expensive. In order to minimize computational time, whilst obtaining a robust understanding of model behaviour, metamodelling approaches are widely used in the literature in the theory and design of computer experiments (Santner et al. 2003; Kleijnen 1995). A metamodel fits a model to the experimental data generated from the simulation model. We use two classes of metamodels: 1) regression metamodels; 2) Gaussian process emulators. For our simulation model, both approaches provide similar results in terms of highlighting which parameters are influential for each of the three model outcomes of interest. Given the good fit provided by both methods (see cross-validation below), and the similarities in the sensitivity analysis results, we draw on Grow (forthcoming) who recommends the use of regression metamodels for simulation approaches as these are widely used by and understood by social scientists. Consequently, we report results from the regression-based analysis of variance in the main text of the article.

**Experimental Design**

For sensitivity analysis of the model across the minimum and maximum theoretically plausible range of the parameter values [see Table 2 in the main article], we run the model at 224 points of a maximim-distance latin hypercube sample (LHS). We repeat the simulation 5 times at a randomly-chosen subset of 44 of these points resulting in a total sample of 400 points. LHS designs have good space-filling properties, perform better than random samples, and have been shown to perform well with regression metamodels (Santner et al. 2003, 158) as well as Gaussian process emulators (Kennedy and O’Hagan 2001). According to standard practice in the literature, we standardize each input parameter to between 0 and 1 to enable comparability for coefficients.

**Regression Metamodel**

Table S1 reports the coefficients from the best fit regression metamodel for the three outcomes of interest from the simulation. Incorporating higher order (above quadratic terms) does not improve model fit. For each of the three outcomes, the R-squared and adjusted R-squared values indicate a robust fit (above 75% in each case) to enable sensitivity analysis conclusions on the basis of the regression metamodel. These regression metamodels highlight how the measure of model fit, the mean SRB in 1990 and the mean maximum SRB respond to different parameters in the simulation. A higher value of the model fit measure indicates worse fit between the simulated and UN estimates of SRBs. The negative signs on the coefficients in the column ‘model fit measure’ in Table S1 indicate that higher values of σ, β (the readiness to abort parameters for parity 1+ and parity 0 respectively) improve model fit for South Korea. As expected, these models highlight that higher values of the readiness to abort parameters predict increased SRB in 1990 as well as maximum SRB levels in the simulation model. SRB levels, in 1990 and the maximum levels attained by the model, are lower with slower rates of technology diffusion, ⍴, and show a negative quadratic shape with Φ, the inflection point for technology diffusion. The analysis of variance (ANOVA) quantifying how the variance in model output is attributable to different parameters are summarized in Table 4 in the main article.

**Gaussian Process Emulators**

In addition to the regression metamodel, we also fit a Gaussian process emulator, a flexible class of metamodels developed for the statistical analysis of computer models, to perform sensitivity analysis. Bijak et al. (2013) apply Gaussian process emulators to a re-implementation of one of the most well-known applications of agent-based modelling in demography, the ‘wedding ring’ model for marriage developed by BIllari et al. (2007). The statistical theory for emulation is outlined in Kennedy and O’Hagan (2001), Oakley and O’Hagan (2002), Oakley and O’Hagan (2006), and O’Hagan (2006). Bijak et al. (2013) also provide an in-depth account of the applications of emulation for demographic uses, including an example of sensitivity analysis using an emulator.

The input data **D** to which the emulator is fitted come from the experimental sample (Latin Hypercube sample) also used in the regression metamodel. **D** contains data from a set of **N** experimental points (in our case, the 224 unique points from the LHS). Let f(.) denote the simulator. A Gaussian Process emulator can be defined as (Oakley and O’Hagan 2002):

$\left[ f\left( . \right) | \theta,\sigma,R \right]\sim N[m\left( \cdot\right),\sigma^{2}c\left( \cdot,\cdot\right)]$ (2)

where, m denotes the mean of the process that is modelled through a regression model of x, *h(x)*, with coefficients $\theta$such that for every output $f\left( x \right), m\left( \cdot\right)={h\left( \cdot\right)}^{T}\theta$. $c\left( \cdot,\cdot\right)$denotes the correlation matrix based on a roughness matrix $R = diag\left( r_{1,}\ldots r_{n} \right)$which captures the responsiveness of the emulator to changes in particular inputs. A ‘nugget’ term to account for simulation stochasticity of the emulator can be incorporated in the estimation of the mean and covariance matrix. If assumptions about the prior distributions of the parameters of the emulator are included, a full Bayesian approach for uncertainty and sensitivity analysis can be applied to obtain a posterior distribution of *f* given D. While the full range of analysis that can be carried out with the emulator particularly with respect to using the model for prediction purposes and uncertainty analysis are wide-ranging, our immediate focus is on building emulators to perform a variance-based sensitivity analysis. For the purpose of our sensitivity analysis, we standardize the input parameters from our simulation model (α, γ, σ, β, φ, ρ) between 0 and 1 and assume them to be described by a uniform distribution: unif(0, 1). Since the minimum and maximum values of already assume a range of theoretically plausible values, our assumption of uniform priors gives equal probability to values within this theoretically plausible range in the absence of other information that would be helpful to reduce uncertainty.

The output variance percentages for the three outputs derived fitting an emulator separately to each are summarized in Table S2. These variance percentages denote how much of the variance in the output is attributable to different parameter combinations. It should be noted that the denominator for these variance-based sensitivity measured from the emulator take the proportion of the variance explained by the inputs, given input distributions, that is, E[Var(f(x))] where f(.) is the simulator. This should theoretically sum to 1 and is thus not directly comparable to an R-squared measure from the regression metamodel. In Table S2 when the total < 1, this implies there are other interaction terms accounting for the variation that were not explicitly estimated. In contrast, in the regression-based ANOVA analysis (see Table 4, main article) the denominator is the total variance in the data, with the variance unexplained captured in the residual variance term.

The model fit measure is especially sensitive to φ, the inflection point for technology diffusion, followed the readiness to abort parameters of σ and β. For the SRB 1990 output measure, the technology diffusion parameters matter, with φ again explaining a sizeable fraction of the variance followed by ρ, the rate of technology diffusion, and σ, the readiness to abort parameter at parity 1 and above. The maximum SRB is most sensitive to σ and β, the readiness to abort parameters. These conclusions concur with those reported from the analysis of variance (ANOVA) in the main text of the paper.

**Metamodel Cross-validation**

For both the regression metamodels and the emulators, we test how well the metamodels compare with the simulator at a sample of 88 input combination takes from outside the input LHS (training) sample. We compare metamodel predictions with the simulator’s output at these points. We calculate the cross-validation root mean squared error (RMSE), which measures the average error between the simulator and the metamodel at a given combination of parameters, and normalized root mean squared error, which measures how much (expressed as %) of the input range (from the LHS data **D**) the average error spans. A normalized RMSE of 0.07 would indicate that the average error spanned 7% of the range of values for that outcome in the input sample. These are reported in Table S3. The RMSE, particularly for the fit measure, indicate that the metamodels do reasonably well at predicting the model fit measure, the SRB at 1990, and maximum SRB at different input points, with the average error within ~ 10% (normalized RMSE < 0.1 except in max SRB) of the input sample range. Other than for the measure of model fit, where the emulator performs better than the regression metamodel, the cross-validation performance for both metamodels are similar.

REFERENCES

Bijak, J., J. Hilton, E. Silverman, and V. D. Cao (2013). Reforging the Wedding Ring: Exploring a semi-artificial model of population for the United Kingdom with Gaussian process emulators. Demographic Research 29(27), 729–766.

Billari, F. C., A. Prskawetz, B. Aparicio Diaz, and T. Fent (2007). The “Wedding- Ring”: An agent-based marriage model based on social interaction. Demographic Research 17(3), 59–82.

A. Grow (2016) Regression Metamodels for Sensitivity Analysis in Agent-Based Computational Demography: A Case Study of Educational Assortative Mating. In: A. Grow & J. Van Bavel (Eds.), Agent-Based Modelling in Population Studies - Concepts, Methods, and Applications. Springer (forthcoming).

Kennedy, M. (2004). Description of the Gaussian process model used in GEM-SA. Software manual [electronic resource]. Sheffield: Centre for Terrestrial Carbon Dynamics, University of Sheffield. [http://ctcd.group.shef.ac.uk/gem.html] (retrieved on 23/05/2012).

Kennedy, M.C. and O‟Hagan, A. (2001). Bayesian calibration of computer models. *Journal of the Royal Statistical Society, Series B* 63(3): 425–464. doi:10.1111/ 1467-9868.00294.

Kleijnen, J. P. (1995). Verification and validation of simulation models. *European Journal of Operational Research* 82(1), 145–162.

Oakley, J. and O‟Hagan, A. (2002). Bayesian inference for the uncertainty distribution of computer model outputs. *Biometrika* 89(4): 769–784. doi:10.1093/biomet/ 89.4.769.

Oakley, J. and O‟Hagan, A. (2004). Probabilistic sensitivity analysis of complex models: A Bayesian approach. *Journal of the Royal Statistical Society, Series B* 66(3): 751–769. doi:10.1111/j.1467-9868.2004.05304.x.

Santner, T. J., B. J. Williams, and W. I. Notz (2003). The design and analysis of computer experiments. Springer-Verlag New York.

TABLES

| Parameters | Model Fit Measure | SRB 1990 | Maximum SRB |
| --- | --- | --- | --- |
|  | *b* | *b* | *b* |
| ⍺ | -0.61 | 2.14 | -0.33 |
| ɣ | 1.37* | -1.1 | 0.9 |
| σ | -7.17*** | 17.11*** | 11.78*** |
| β | -3.28*** | 0.26 | 4.93 |
| Φ | 0.69 | 0.94 | 1.49 |
| ⍴ | 1.7* | -17.19*** | -5.02 |
| Ɣ^2^ | -2.71*** | 2.67 | 0.89 |
| σ^2^ | 3.54*** | -10.9*** | -5.74 |
| β^2^ | 0.71 | 2.73* | -3.31 |
| Φ^2^ | 3.22*** | -5.57*** | -1.32 |
| ⍴^2^ | -2.04*** | 9.59*** | 2.16 |
| ⍴ . Φ | 3.72*** | 14.28*** | 4.4 |
| σ . Φ | -2.68*** | 1.05 | -2.55 |
| β . Φ | -0.76 | -0.91 | -1.66 |
| σ . ⍴ | 0.91 | 7.42*** | 2.28 |
| β . ⍴ | -1.84*** | 4.14* | 0.11 |
| σ.β | 5.62*** | 0.15 | 1.18 |
| ɣ . σ | 3.52*** | 2.51* | 4.11*** |
| ɣ . ⍴ | -0.92 | 5.62** | 1.18 |
| ɣ . Φ | -0.83 | 0.87 | -0.92 |
| ⍺ . Β | 2.29*** | -2.59* | 0.89 |
| β . Φ | 1.61*** |  |  |
| ⍺ . ⍴ | 1.11** | 0.45 | 1.88 |
| ⍺ . Φ | -1.67*** | -1.66 | -1.16 |
| ⍺ . Ɣ |  | 0.09 | 0.43 |
| σ . Φ . ⍴ |  | -23.9*** | -5.25 |
| β . ⍴ . Φ |  | -11.69** | -4.04 |
| ɣ . ⍴ . Φ |  | -17.4*** | -3.71 |
| Intercept | 3.93*** | 106.99*** | 110.39*** |
| Multiple R-squared | 0.7739 | 0.8701 | 0.7916 |
| Adjusted R-squared | 0.7602 | 0.8605 | 0.7731 |
|  |  |  |  |
| * p < 0.05 | ** p < 0.01 | *** p < 0.001 |  |

Table S1: Standardized coefficients *b* from best-fit regression metamodels predicting three outcomes from simulation experiments: 1) model fit measure; 2) SRB 1990; 3) maximum SRB.

|  |  | Variance (%) | |
| --- | --- | --- | --- |
| Parameter | Model Fit Measure | SRB 1990 | Maximum SRB |
| ⍺ | 0.13 | 0.27 | 1.18 |
| ɣ | 5.01 | 4.13 | 14.33 |
| σ | 5.32 | 13.81 | 47.68 |
| β | 3.46 | 6.72 | 23.12 |
| Φ | 37.39 | 48.23 | 7.43 |
| ⍴ | 4.86 | 6.42 | 1.02 |
| ⍺ . Ɣ | 0.11 | 0.01 | 0.05 |
| ⍺ . σ | 0.33 | 0.03 | 0.11 |
| ⍺ . Β | 0.05 | 0.02 | 0.03 |
| ⍺ . Φ | 0.53 | 0.18 | 0.03 |
| ⍺ . ⍴ | 0.06 | 0.04 | 0.02 |
| ɣ . σ | 4.9 | 0.54 | 1.56 |
| ɣ . Β | 2.21 | 0.15 | 0.11 |
| ɣ . Φ | 2.88 | 1.1 | 0.42 |
| ɣ . ⍴ | 0.36 | 0.54 | 0.06 |
| σ.β | 11.05 | 0.11 | 0.23 |
| σ . Φ | 4 | 5.32 | 1.34 |
| σ . ⍴ | 0.45 | 0.31 | 0.22 |
| β . Φ | 1.3 | 2.72 | 0.56 |
| β . ⍴ | 0.2 | 0.28 | 0.17 |
| ⍴ . Φ | 5.78 | 5.75 | 0.11 |
| Total | 90.38 | 96.68 | 99.78 |

Table S2: Variance (%) explained by different parameters and their interaction for three outcomes measures: 1) model fit measure; 2) SRB in 1990; 3) maximum SRB as estimated from the gaussian process emulator.

| Output | Cross-validation Statistic | Regression Metamodel | Emulator |
| --- | --- | --- | --- |
| Model Fit Measure | RMSE | 0.64 | 0.25 |
|  | Normalized RMSE | 0.1 | 0.04 |
|  |  |  |  |
| SRB 1990 | RMSE | 1.51 | 2.52 |
|  | Normalized RMSE | 0.07 | 0.09 |
|  |  |  |  |
| Maximum SRB | RMSE | 1.88 | 1.87 |
|  | Normalized RMSE | 0.12 | 0.12 |

Table S3: Summary of cross-validation statistics indicating metamodel fit with simulated data for two classes of metamodels: 1) regression metamodel; 2) emulator.
